# Mid-Term Outcomes of the Viabahn Balloon-Expandable Endoprosthesis as Bridging Stent Graft for Fenestrated and Branched Endovascular Aortic Repair

**DOI:** 10.1177/15266028241300005

**Published:** 2024-11-22

**Authors:** Kaj O. Kappe, Samira E.M. van Knippenberg, Bich L. Tran, Rutger J. Lely, Bram B. van der Meijs, Jan D. Blankensteijn, Johanna H. Nederhoed, Ron Balm, Vincent Jongkind, Arjan W.J. Hoksbergen, Kak Khee Yeung

**Affiliations:** 1 Department of Surgery, Amsterdam University Medical Centers, Location Vrije Universiteit, Amsterdam, The Netherlands; 2 Amsterdam Cardiovascular Sciences, Amsterdam, The Netherlands; 3 Department of Surgery, Amsterdam University Medical Centers, Location University of Amsterdam, Amsterdam, The Netherlands; 4 Department of Radiology and Nuclear Medicine, Amsterdam University Medical Centers, Location Vrije Universiteit, Amsterdam, The Netherlands

**Keywords:** aortic aneurysm, endovascular aneurysm repair, endoprosthesis, fenestrated endovascular aneurysm repair, branched endovascular aneurysm repair, bridging stent graft, Viabahn VBX

## Abstract

**Purpose:**

Bridging stent grafts (BSG) implanted during fenestrated and branched endovascular aortic repair (F/B-EVAR) are crucial for the successful exclusion of thoracoabdominal and complex abdominal aortic aneurysms (AAA). The aim of this study was to analyze the outcomes of the Gore Viabahn VBX stent graft as BSG for renal and visceral target vessels during F/B-EVAR.

**Materials and Methods:**

All consecutive patients undergoing F/B-EVAR for thoracoabdominal or complex AAAs from January 2019 to May 2023 who were treated with at least 1 VBX stent graft as BSG were included. Procedural, radiological, and follow-up data of the included patients were retrospectively reviewed. Primary outcome of the study was technical success of VBX stent graft implantation. Secondary endpoints were VBX-related adverse events, target vessel instability, endoleaks, and overall survival.

**Results:**

A total of 273 VBX stent grafts were implanted in 263 target vessels in 38 FEVAR, 46 BEVAR, and 3 F/B-EVAR (combined design) stent grafts in 87 patients (74.7% male; mean age, 73.6 ± 7.0 years). Technical success of VBX stent graft implantation was 97.5% with 273 successful implantations in 280 attempts. The VBX-related secondary endpoints were evaluated for 269 VBX stent grafts in 259 target vessels. Target vessel designs included 107 fenestrations (41.3%), 82 outer-branches (31.7%), and 70 inner-branches (27.0%). Freedom from VBX-related adverse events at 12 months postoperatively was 96.6% (95% CI: 92.9%-100%) for target vessels with a fenestration and 93.6% (95% CI: 89.4%-98.0%) for target vessels with a branch. Freedom from target vessel instability at 12 months postoperatively for fenestrations and branches was 98.1% (95% CI: 94.4%-100%) and 97.6% (95% CI: 94.9%-100%) respectively. A total of 9 (3.5%) VBX-related endoleaks were detected during follow-up. Overall survival of all treated patients was 86.7% (95% CI: 79.1%-94.9%) at a median follow-up of 14 months.

**Conclusion:**

The VBX stent graft shows an excellent performance as a BSG in F/B-EVAR. The VBX stent graft has a high technical implantation success and shows a high mid-term freedom from stent graft–related adverse events and target vessel instability for both target vessels with a fenestration and a branch. Long-term follow-up data of the performance of the VBX stent graft are to be awaited.

**Clinical Impact:**

This study evaluated the outcomes of the Gore Viabahn VBX stent graft as a bridging stent graft (BSG) for renal and visceral target vessels. It is important to evaluate the performance of such recently introduced stent grafts as these are essentially used in procedures outside of the Instructions for Use. This comprehensive analysis of the VBX stent graft as a BSG adds to the evidence of the performance of this stent graft as a BSG.

## Introduction

Fenestrated and branched endovascular aortic repair (F/B-EVAR) is a well-accepted minimally-invasive approach for the treatment of thoracoabdominal and complex abdominal aortic aneurysms (AAA). This minimally-invasive approach has enabled the possibility to treat high-risk patients, that is, an older patient population with more comorbidities, with comparable early mortality and perioperative complication rates compared with traditional open surgical repair.^1^

While F/B-EVAR offers the advantage of treating high-risk patients, it is also associated with a higher rate of reinterventions.^2,3^ The majority of reinterventions after complex endovascular repair are related to target vessel complications.^4^ A successful durable exclusion of the aneurysm with F/B-EVAR is therefore heavily dependent on the performance of the bridging stent grafts (BSG) that connect the main stent graft to the target vessels. However, up to this date, there is no dedicated BSG for the use in F/B-EVAR and all currently implanted BSGs are used off-label.

The Gore VIABAHN VBX Balloon-Expandable Endoprosthesis (WL Gore and Associates, Flagstaff, Arizona) is one of the stent grafts used off-label as a BSG in F/B-EVAR. The advantage of the VBX stent graft is its high flexibility on the balloon catheter which allows deployment through (steerable) sheaths advanced via femoral access only. Femoral access, when compared with upper extremity access, is associated with a significant decrease in operative and fluoroscopy time and a decrease in neurological complications such as stroke.^5,6^

As the performance of the VBX stent graft as a BSG has been sparsely studied, the aim of this study was to report the outcomes of its use as a BSG for renal and visceral target vessels in F/B-EVAR procedures.

## Methods

### Study Design and Patient Population

This study is a single-center retrospective cohort study performed in a tertiary referral center. All consecutive patients undergoing F/B-EVAR with at least one VBX stent graft implanted as a BSG from January 2019 to May 2023 were selected from a prospectively maintained database. Patients treated for a thoracoabdominal or complex AAA, both elective and acute cases, were included. The choice of the length, diameter, number, and type of BSG was made by the treating vascular surgeons based on the preoperative planning and intraoperative findings.

### Data Collection

Data of the included patients were collected retrospectively from the patients’ electronic medical records. The study was approved by the local medical ethical review board. The collected data included baseline demographics, procedural data, clinical follow-up data, and computed tomography angiographic (CTA) images. Clinical follow-up data were collected until the last out-patient visit or telephone consultation and imaging was collected up to the most recent CTA examination. The CTA examination is performed 4 to 6 weeks postoperatively per protocol, at discretion of the surgeon at 6 months, and per protocol at 1 year and annually thereafter. For optimal BSG positioning and deployment, pre- and post-deployment selective angiograms are performed for every target vessel. Finally, a completion angiogram of the complete aortic reconstruction is performed at the end of the procedure.

### Study Endpoints

The primary endpoint of the study was technical success of implantation of the VBX BSG in the target vessel. Technical success of implantation was defined as successful introduction and deployment of the VBX BSG in the target vessel. Intentional placement of VBX BSG in a separate procedure, in the context of temporary sac perfusion to minimize the risk of spinal cord ischemia, was considered as the first attempt of VBX implantation and deployment.

Secondary endpoints of the study included VBX-related adverse events during follow-up, target vessel instability, and overall survival. The VBX-related adverse events were defined as kinking, stenosis, occlusion, or migration of the VBX stent graft or a type 1c or type 3 endoleak at the site of the VBX stent graft. Target vessel instability, according to the Society of Vascular Surgery (SVS) Reporting Standards, is a subgroup of VBX-related adverse events leading to death, rupture, or secondary interventions.^7^ Target vessels with a VBX stent graft accompanied by a BSG from a different manufacturer were included in the analysis as well. In addition, type 1c and type 3 endoleaks were described separately.

Analysis of VBX-related adverse events was based on routine CTA imaging during follow-up. These analyses only included patients with CTA imaging available during follow-up. The radiological reports of the acquired CTA imaging were screened for potential VBX-related adverse events. All CTA images with possible VBX-related adverse events were independently re-evaluated by 2 experienced endovascular surgeons. Discrepancies in the judgment of the potential VBX-related adverse events by the 2 endovascular surgeons were discussed in a consensus meeting with the interventional radiologists.

### Statistical Analysis

Continuous variables with a normal distribution were expressed as mean ± standard deviation and as median and interquartile range (IQR) for skewed variables. Categorical data were expressed as numbers and percentages. Kaplan-Meier curves were used to analyze time-dependent outcomes in this study. A per target vessel analysis was performed for the VBX-related adverse events and target vessel instability. Statistical analyses were performed using SPSS version 28 (IBM Corp, Armonk, New York) and R software (R Core Team, version 4.2.1, Vienna, Austria).

## Results

### Patient Cohort

**Figure 1.**
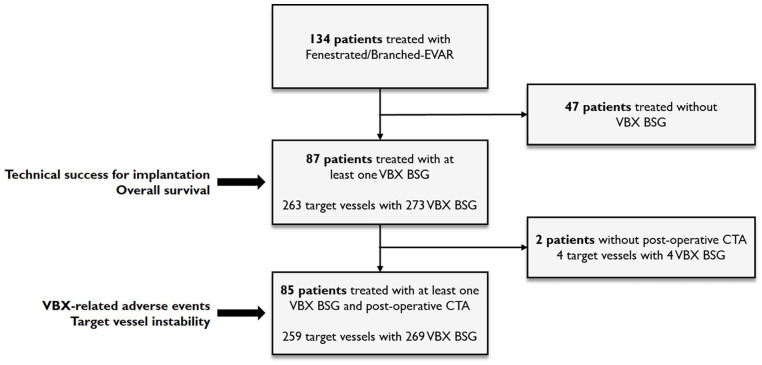
Flowchart illustrating the patient selection and cohorts for evaluation of the primary endpoint and secondary endpoints.

A total of 87 patients (mean age 73.6 ± 7.0 years), comprising 65 males (74.7%) and 22 females (25.3%), were treated with F/B-EVAR and received at least 1 VBX stent graft as BSG (Figure 1). Two patients were treated for a Crawford type I (2.3%), 23 for a Crawford type II (26.4%), 3 for a Crawford type III (3.4%), 13 for a Crawford type IV (14.9%), 2 for a pararenal (2.3%), and 44 for a juxtarenal (50.6%) aortic aneurysms with a mean diameter of 65.9 ± 11.9 mm. Eighty-one patients were asymptomatic (93.1%), 3 patients were symptomatic (3.4%), and 3 patients were treated for a ruptured aneurysm (3.4%). Twenty-one patients had a prior open aortic repair (24.1%) and 34 patients had a previous endovascular aortic repair (39.1%). All baseline characteristics and aneurysm characteristics are shown in Tables 1 and 2.

**Table 1.** Baseline demographics of all patients.

Baseline characteristics	
*Patient characteristics*	N = 87
Age, y	73.6 ± 7.0
Male sex	65 (74.7)
*Medical history*	
Hypertension	71 (81.6)
Coronary artery disease^a^	34 (39.1)
Congestive heart failure	14 (16.1)
Arrhythmia	29 (33.3)
Chronic obstructive pulmonary disease	21 (24.1)
Renal disease^b^	34 (41.5)^c^
Dialysis	0 (0)
Peripheral artery disease	17 (19.5)
Diabetes mellitus	15 (17.2)
Stroke or TIA	18 (20.7)
Tobacco use (past and present)	77 (88.5)
(Previous) Malignancy	23 (26.4)
*Preoperative laboratory values*	
Baseline hemoglobin, *mmol/L*	7.9 ± 1.2
Baseline platelets, *10e9/L*	224.1 ± 68.8
Creatinine, *μmol/L*	104.3 ± 42.1
eGFR, *mL/min/1.73 m^2^*	63.1 ± 20.2
*ASA classification*	
2	15 (17.2)
3	61 (70.1)
4	11 (12.6)
*Routine medication use*	
Lipid-modifying agent	57 (65.5)
Antihypertensive drugs	72 (82.8)
Antithrombotic drugs	23 (26.4)
Antiplatelet therapy	65 (74.7)
Anti-diabetic drugs	15 (17.2)

Data are represented as mean ± standard deviation or as No. (%).

Abbreviations: TIA, transient ischemic attack; eGFR, estimated glomerular filtration rate; ASA, American Society of Anesthesiology.

a Clinical symptoms related to atherosclerotic plaques of the coronary arteries requiring medical intervention/surgery, including myocardial infarction and angina pectoris.

b Renal disease is defined as stages 3a–5 (an estimated glomerular filtration rate of <60 mL/min/1.73 m^2^) as defined by the National Kidney Foundation.

c Missing values in 5 patients.

**Table 2.** Overview of the aneurysm characteristics and stent graft configurations.

Aneurysm characteristics	
*Clinical presentation*	
Asymptomatic	81 (93.1)
Symptomatic	3 (3.4)
Ruptured	3 (3.4)
*Aneurysm classification*	
Crawford I	2 (2.3)
Crawford II	23 (26.4)
Crawford III	3 (3.4)
Crawford IV	13 (14.9)
Pararenal	2 (2.3))
Juxtarenal	44 (50.6)
*Etiologic classification*	
Degenerative	75 (86.2)
Post-dissection aneurysm	8 (9.2)
Mycotic	3 (3.4)
Connective tissue disorder	1 (1.1)
*Previous aortic repair*	
Endovascular aneurysm repair	34 (39.1)
Open surgical repair	21 (24.1)
Maximum aortic diameter, mm	65.9 ± 11.9
Stent graft configuration	
*Manufacturer*	
Cook	52 (59.8)
Jotec/Artivion	21 (24.1)
Terumo	14 (16.1)
*Type of stent graft*	
Fenestrated EVAR	38 (43.7)
Inner-branched EVAR	20 (23.0)
Outer-branched EVAR	26 (29.9)
Combined stent graft design	3 (3.4)

Data are represented as mean ± standard deviation or as No. (%).

Abbreviations: EVAR, endovascular aneurysm repair.

Different configurations of the main stent graft were used (Table 2). A fenestrated stent graft was implanted in 38 patients (43.7%), 46 patients received a branched (inner- and outer-branches) stent graft (52.9%), and 3 patients were treated with a combined fenestrated-branched stent graft (3.4%). Procedural characteristics and clinical outcomes of all patients are listed in Supplementary Tables 1 and 2.

### Primary Endpoint

In total, 273 VBX stent grafts were implanted in 263 target vessels (Figure 1). Target vessel designs were divided into 109 fenestrations (41.5%), 84 outer-branches (31.9%), and 70 inner-branches (26.6%). Technical success of implantation of the VBX stent graft as a BSG was 97.5%; 273 out of 280 VBX stent grafts were implanted successfully. Seven VBX stent grafts could not be implanted during the procedure due to unfavorable anatomy and technical reasons. Table 3 provides the reasons for the implant failures.

**Table 3.** VBX implantation failures and resolutions.

Target vessel	Target vessel design	Reason for implant failure	Resolution
CT	Outer-branch	Stenosed CT	Placement of VBX in second procedure due to prolonged first procedure
LRA	Outer-branch	Unfavorable angulation of LRA for femoral approach	Planned second procedure for VBX placement through brachial access. However, interprocedural occlusion of LRA for which placement of Amplatzer plug
CT	Outer-branch	Luxation of VBX out of CT	Placement of Amplatzer plug in CT
LRA	Inner-branch	VBX slid off balloon catheter	Placement of new VBX in LRA during same procedure
LRA	Fenestration	Target vessel blocked by strut of in situ EVAR	None
CT	Outer-branch	Unable to pass VBX through guiding catheter	Placement of new VBX during same procedure
RRA	Fenestration	Target vessel blocked by strut of in situ EVAR	None

Abbreviations: CT, celiac trunk; LRA, left renal artery; RRA, right renal artery.

### Secondary Endpoints

In total, 85 patients had follow-up CTA imaging and were eligible for evaluation of the VBX-related secondary endpoints. In these 85 patients, a total of 269 VBX stent grafts were used as BSGs in 259 target vessels. Target vessel designs were divided into 107 fenestrations (41.3%), 82 outer-branches (31.7%), and 70 inner-branches (27.0%). Ten target vessels received an additional VBX stent graft and 3 target vessels received an additional Advanta V12 stent graft for proximal or distal extension. Supplementary Table 3 illustrates the number of VBX stent grafts implanted as BSG per target vessel. For the implantation of the VBX stent grafts, femoral access was used for 207 (79.9%) target vessels and brachial access for 52 (20.1%) target vessels (see Supplementary Table 4).

**Figure 2.**
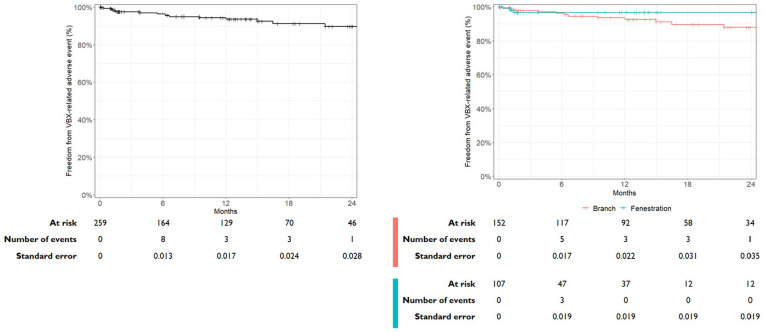
Kaplan-Meier curve illustrating the freedom from any VBX-related adverse event during follow-up for all target vessels and stratified by target vessel design. Two VBX-related adverse events occured after 24 months of follow-up.

Median CTA follow-up in patients eligible for evaluation of the secondary endpoints was 12 months (IQR: 2–20 months). The Kaplan-Meier curve per target vessel illustrating freedom from any VBX-related adverse events during follow-up is shown in Figure 2. At a median CTA follow-up of 12 months, 94.4% (95% confidence interval [CI]: 91.1%–97.7%) of the target vessels were free from any VBX-related adverse event. When stratified by target vessel design, the freedom from VBX-related adverse events for fenestrations was 96.6% (95% CI: 92.9%–100%) and 93.6% (95% CI: 89.4%–98.0%) for branches (combined inner- and outer-branches) (see Figure 2).

**Figure 3.**
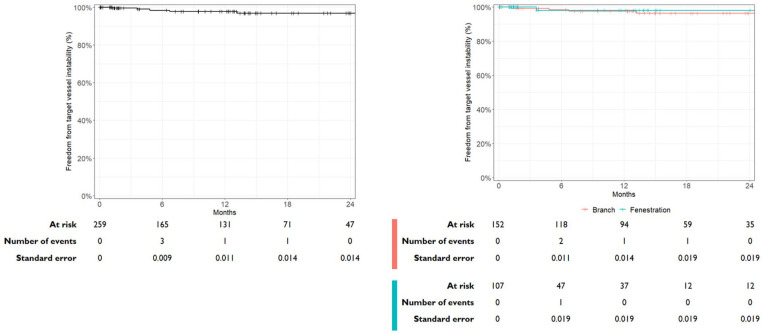
Kaplan-Meier curve illustrating the freedom from target vessel instability during follow-up for all target vessels and stratified by target vessel design. Two target vessel instabilities occured after 24 months of follow-up.

Seven target vessel instabilities were observed during the follow-up period, for which reinterventions were performed: 2 for significant in-stent stenosis of the VBX BSG, 1 for occlusion of the VBX BSG, 1 for tapering of the VBX BSG, 1 for a type 1c endoleak, and 2 for a type 3c endoleak at the site of the VBX BSG. Freedom from target vessel instability at 12 months was estimated at 97.8% (95% CI: 95.6%–100%) (see Figure 3). When stratified by target vessel design, the freedom from target vessel instability for fenestrations was 98.1% (95% CI: 94.4%–100%) and 97.6% (95% CI: 94.9%–100%) for branches, as shown in the Kaplan-Meier curve in Figure 3.

Tables 4 and 5 give an overview of all VBX-related adverse events and target vessel instabilities, and provide the type of secondary intervention or the reason to waive a secondary intervention. The 3 target vessels with an additional Advanta V12 for proximal or distal extension did not show any adverse events.

**Table 4.** VBX-related adverse events during follow-up without reintervention.

Target vessel	Target vessel design	VBX-related adverse event	Reason for no intervention
RRA	Outer-branch	Occlusion VBX BSG	Renogram shows afunctional right kidney
CT	Inner-branch	Kinking + stenosis VBX BSG	Lost to follow-up
RRA	Inner-branch	Type 3c endoleak	No growth of the aneurysm
LRA	Inner-branch	Type 3c endoleak	Previously interpreted differently
CT	Outer-branch	Occlusion VBX BSG	Collateral inflow to CT from SMA
SMA	Outer-branch	Type 3c endoleak	No growth of aneurysm
CT	Outer-branch	In-stent stenosis	Deemed sufficient flow through in-stent stenosis
LRA	Fenestration	Type 3c endoleak	Termination antiplatelet therapy as first approach
LRA	Outer-branch	Type 3c endoleak	Short follow-up
RRA	Fenestration	Type 3c endoleak	Short follow-up

Abbreviations: CT, celiac trunk; SMA, superior mesenteric artery; LRA, left renal artery; RRA, right renal artery.

**Table 5.** Target Vessel Instability (VBX-related adverse events during follow-up requiring reintervention).

Target vessel	Target vessel design	VBX-related adverse event	Type of reintervention
SMA	Outer-branch	Type 3c endoleak	Relining of VBX BSG
RRA	Outer-branch	Tapering	PTA of VBX BSG
LRA	Inner-branch	In-stent stenosis	PTA of VBX BSG
SMA	Inner-branch	Type 1c endoleak	Relining of VBX BSG
LRA	Outer-branch	In-stent stenosis	PTA of VBX BSG
RRA	Fenestration	Type 3c endoleak	PTA + proximal and distal extension by relining of VBX BSG
LRA	Outer-branch	Occlusion	Alteplase in proximal LRA

Abbreviations: CT, celiac trunk; SMA, superior mesenteric artery; LRA, left renal artery; RRA, right renal artery.

**Figure 4.**
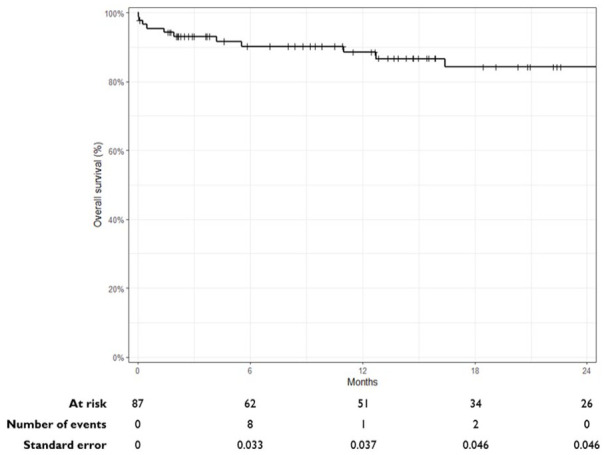
Kaplan-Meier curve illustrating the overall survival of all patients treated with F/B-EVAR. Five deaths occurred after 24 months of follow-up.

The estimated overall survival, as shown in the Kaplan-Meier analysis in Figure 4, of all patients undergoing F/B-EVAR (87 in total) was 86.7% (95% CI: ﻿ 79.1%–94.9%) at a median clinical follow-up of 14 months (IQR: 4–26 months). Nine patients died in the first postoperative year, with none of the deaths related to complications of the VBX stent graft.

The VBX-related endoleaks were observed at the site of 9 (3.5%) target vessels (see Table 6).

**Table 6.** Endoleaks at the site of the target vessels.

Type of endoleak	Location	Type of branch
3c	SMA	Outer-branch
3c	RRA	Inner-branch
1c	SMA	Inner-branch
3c	LRA	Inner-branch
3c	RRA	Fenestration
3c	SMA	Outer-branch
3c	LRA	Fenestration
3c	LRA	Outer-branch
3c	RRA	Fenestration

Abbreviations: CT, celiac trunk; SMA, superior mesenteric artery; LRA, left renal artery; RRA, right renal artery.

## Discussion

The BSGs in F/B-EVAR are the Achilles heel in the design of endovascular solutions for thoracoabdominal and complex abdominal aortic repair, accounting for the majority of reinterventions.^4^ It is essential to assess the performance of recently introduced stent grafts used to bridge the main aortic stent grafts and aortic side branches, as the use of these stent grafts in this specific approach falls outside the Instruction For Use.

This single-center retrospective study investigated the VBX stent graft as BSG in F/B-EVAR and showed that implantation of this specific stent graft has a high technical success of 97.5%. Furthermore, at a median follow-up of 12 months, this study indicates a high freedom from VBX-related adverse events and target vessel instability of 96.6% and 98.1%, respectively, when used in target vessels with a fenestration. In target vessels with a branch, freedom from VBX-related adverse events and target vessel instability was 93.6% and 97.6%, respectively.

Both target vessel instability and VBX-related adverse events are important to report. According to the SVS Reporting Standards, target vessel instability is one of the proposed endpoints to evaluate target vessel–related outcomes during follow-up of F/B-EVAR.^7^ Target vessel instability is defined as a target vessel–related adverse event with a clinical consequence, comprising rupture, death, or a secondary intervention. Our study also evaluated VBX-related adverse events from a more comprehensive perspective, also reporting the VBX-related adverse events without a clinical consequence, for example, a subclinical occlusion or stenosis, or a conservatively treated endoleak. In our cohort, less than half of the identified VBX-related adverse events required a secondary intervention, with none of the adverse events leading to death or rupture. However, VBX-related adverse events that do not lead to death, rupture, or secondary intervention are still of importance and worth reporting as these may require a modified follow-up. The results of our study therefore highlight that reporting only target vessel instability would underreport overall target vessel complications, and suggest a more beneficial performance of the BSG.

The high freedom from target vessel instability in our cohort is consistent with previous studies evaluating VBX as BSG. Previous studies by Torsello et al^8^ and Motta et al^9^ demonstrated a freedom from target vessel instability in target vessels with a branch of 95.8% and 95.6% at a median follow-up of 18 and 24 months, respectively. The subgroup of target vessels with a branch in our cohort shows a freedom from target vessel instability of 97.5% at 12 months postoperatively. Freedom from target vessel instability for target vessels with a fenestration in our cohort at 12 months postoperatively was 98.1%. The largest available series in the literature evaluating target vessel instability in 48 target vessels with a fenestration targeted with a VBX BSG shows a freedom from target vessel instability of 92.3% at 6 months follow-up, with the remark that only renal target vessels were evaluated in a per patient analysis.^10^ The freedom from target vessel instability of the VBX as a BSG in our study (fenestrations 98.1%, branches 97.6%) is comparable with the results of the Advanta V12 as a BSG (fenestrations 98.9%, branches 96.4%) at 12 months postoperatively.^11^ In our study, the definition of target vessel instability was in accordance with the SVS reporting standards.^7^ However, it should be noted that the definitions of target vessel instability have minor variations between available studies in the literature.^9,10^

Target vessel–related adverse events and target vessel instability are influenced by multiple factors. These include aneurysm morphology, the design of the main stent graft in combination with the used BSG, and the suitability of the main stent graft for the aneurysm morphology, that is, a custom-made or off-the-shelf device. To illustrate, a large single-center study by Katsargyris et al^11^ demonstrated a significantly lower freedom from target vessel instability for branches compared with fenestrations. Piazza et al^12^ reported a similar freedom from target vessel instability for BSG in inner-branch and outer-branch configurations. Numerically, more VBX-related adverse events and target vessel instabilities were observed for branched main-graft configurations compared with fenestrated main-grafts in this study. However, the current study lacks the power to further analyze the influence of the main-graft configuration.

While endoleaks at the site of the bridging stents are the major reason for BSG-related reinterventions, characterizing and determining the fate of an endoleak remains a clinical challenge.^4,13^ Squizzato et al^13^ and Kärkkäinnen et al^14^ demonstrated that a significant number of endoleaks detected on the first postoperative CTA resolve spontaneously during follow-up. As our study is restricted by the limited follow-up period, the clinical consequences of type 1c or type 3c endoleaks on the first postoperative CTA and without a subsequent CTA remain uncertain.

Overall survival of the treated patients of 86.7% at a median follow-up of 14 months in our cohort is in line with other studies.^8,15,16^ In our cohort, there were 2 aneurysm-related deaths and 2 procedure-related deaths in the first postoperative year. However, none of these deaths were directly attributed to the performance of the VBX stent graft as a BSG.

The main limitation of this study is the retrospective design. The radiological reports were retrospectively screened for possible VBX-related adverse events. Ideally, initial evaluation of the CTA is performed by 2 independent observers. However, only the CTAs with possible VBX-related adverse events in the radiological report were included for re-evaluation by 2 experienced endovascular surgeons. This may impose missed VBX-related adverse events, though it would only include those without clinical consequences. Second, our study did not compare the performance of the VBX BSG between target vessels with a fenestration and branch as the study was not designed for this purpose. Furthermore, long-term outcomes of the VBX stent graft as a bridging stent remain unknown due to the limited follow-up period in this study.

## Conclusion

This retrospective study showed an excellent performance of the VBX stent graft as a BSG in F/B-EVAR in patients treated for a thoracoabdominal or complex aortic aneurysm. The VBX stent graft can be implanted with a high technical success. A high mid-term freedom from VBX-related adverse event and target vessel instability was observed. Long-term outcomes of the VBX BSG in F/B-EVAR are to be awaited.

## Supplemental Material

sj-docx-1-jet-10.1177_15266028241300005 – Supplemental material for Mid-Term Outcomes of the Viabahn Balloon-Expandable Endoprosthesis as Bridging Stent Graft for Fenestrated and Branched Endovascular Aortic Repair Supplemental material, sj-docx-1-jet-10.1177_15266028241300005 for Mid-Term Outcomes of the Viabahn Balloon-Expandable Endoprosthesis as Bridging Stent Graft for Fenestrated and Branched Endovascular Aortic Repair by Kaj O. Kappe, Samira E.M. van Knippenberg, Bich L. Tran, Rutger J. Lely, Bram B. van der Meijs, Jan D. Blankensteijn, Johanna H. Nederhoed, Ron Balm, Vincent Jongkind, Arjan W.J. Hoksbergen and Kak Khee Yeung in Journal of Endovascular Therapy

sj-docx-2-jet-10.1177_15266028241300005 – Supplemental material for Mid-Term Outcomes of the Viabahn Balloon-Expandable Endoprosthesis as Bridging Stent Graft for Fenestrated and Branched Endovascular Aortic Repair Supplemental material, sj-docx-2-jet-10.1177_15266028241300005 for Mid-Term Outcomes of the Viabahn Balloon-Expandable Endoprosthesis as Bridging Stent Graft for Fenestrated and Branched Endovascular Aortic Repair by Kaj O. Kappe, Samira E.M. van Knippenberg, Bich L. Tran, Rutger J. Lely, Bram B. van der Meijs, Jan D. Blankensteijn, Johanna H. Nederhoed, Ron Balm, Vincent Jongkind, Arjan W.J. Hoksbergen and Kak Khee Yeung in Journal of Endovascular Therapy

sj-docx-3-jet-10.1177_15266028241300005 – Supplemental material for Mid-Term Outcomes of the Viabahn Balloon-Expandable Endoprosthesis as Bridging Stent Graft for Fenestrated and Branched Endovascular Aortic Repair Supplemental material, sj-docx-3-jet-10.1177_15266028241300005 for Mid-Term Outcomes of the Viabahn Balloon-Expandable Endoprosthesis as Bridging Stent Graft for Fenestrated and Branched Endovascular Aortic Repair by Kaj O. Kappe, Samira E.M. van Knippenberg, Bich L. Tran, Rutger J. Lely, Bram B. van der Meijs, Jan D. Blankensteijn, Johanna H. Nederhoed, Ron Balm, Vincent Jongkind, Arjan W.J. Hoksbergen and Kak Khee Yeung in Journal of Endovascular Therapy

sj-docx-4-jet-10.1177_15266028241300005 – Supplemental material for Mid-Term Outcomes of the Viabahn Balloon-Expandable Endoprosthesis as Bridging Stent Graft for Fenestrated and Branched Endovascular Aortic Repair Supplemental material, sj-docx-4-jet-10.1177_15266028241300005 for Mid-Term Outcomes of the Viabahn Balloon-Expandable Endoprosthesis as Bridging Stent Graft for Fenestrated and Branched Endovascular Aortic Repair by Kaj O. Kappe, Samira E.M. van Knippenberg, Bich L. Tran, Rutger J. Lely, Bram B. van der Meijs, Jan D. Blankensteijn, Johanna H. Nederhoed, Ron Balm, Vincent Jongkind, Arjan W.J. Hoksbergen and Kak Khee Yeung in Journal of Endovascular Therapy
