# Supplementary material for: Mid-Term Outcomes of the Viabahn Balloon-Expandable Endoprosthesis as Bridging Stent Graft for Fenestrated and Branched Endovascular Aortic Repair
Source: J Endovasc Ther. 2024 Nov 22;33(3):1354–62. doi: 10.1177/15266028241300005 (PMC13172123; doi:10.1177/15266028241300005)
Supplement: sj-docx-1-jet-10.1177_15266028241300005 – Supplemental material for Mid-Term Outcomes of the Viabahn Balloon-Expandable Endoprosthesis as Bridging Stent Graft for Fenestrated and Branched Endovascular Aortic Repair [file sj-docx-1-jet-10.1177_15266028241300005.docx]

**Supplementary table 1: Procedural characteristics**

| **Procedural characteristics** |  |
| --- | --- |
| Procedure time, *min* | 263.3 ± 95.0 |
| Contrast volume, *mL* | 148.3 ± 59.7 |
| Fluoroscopy time, *min* | 92.0 ± 44.2 |
| Post-op eGFR, *mL/min/1.73m2* | 59.60 ± 20.8 |

**Supplementary table 1:** Overview of the procedural characteristics. Data are represented as mean (standard deviation).
