# Supplementary material for: Mid-Term Outcomes of the Viabahn Balloon-Expandable Endoprosthesis as Bridging Stent Graft for Fenestrated and Branched Endovascular Aortic Repair
Source: J Endovasc Ther. 2024 Nov 22;33(3):1354–62. doi: 10.1177/15266028241300005 (PMC13172123; doi:10.1177/15266028241300005)
Supplement: sj-docx-2-jet-10.1177_15266028241300005 – Supplemental material for Mid-Term Outcomes of the Viabahn Balloon-Expandable Endoprosthesis as Bridging Stent Graft for Fenestrated and Branched Endovascular Aortic Repair [file sj-docx-2-jet-10.1177_15266028241300005.docx]

**Supplementary table 2: Clinical outcomes**

| **Clinical outcomes** |  |
| --- | --- |
| Hospital stay, days, IQR | 3 [2-6] |
| ICU stay, days, IQR | 0 [0-1] |
| Clinical follow-up, months, median [IQR] | 14 [4-26] |
| Death | 16 (18.4) |
| < 30 days or during hospitalization | 5 (5.7) |
| Cause of death |  |
| Aneurysm-related | 4 (4.6) |
| Cardiovascular | 2 (2.3) |
| Malignancy | 1 (1.1) |
| Sepsis | 1 (1.1) |
| Unknown | 8 (9.2) |
| Short-term outcomes (< 30 days or during hospitalization) |  |
| Myocardial infarction | 2 (2.3) |
| Stroke | 2 (2.3) |
| Spinal cord ischemia | 4 (4.6) |
| Symptoms after discharge or until death | 2 (2.3) |
| Acute kidney injury leading to dialysis | 1 (1.1) |
| Bowel ischemia | 1 (1.1) |
| Access site complications leading to intervention | 5 (5.7) |
| Graft infection | 1 (1.1) |

**Supplementary table 2:** Clinical outcomes after index procedure. None of the clinical complications occurred after a second procedure.
