# Supplementary material for: Mid-Term Outcomes of the Viabahn Balloon-Expandable Endoprosthesis as Bridging Stent Graft for Fenestrated and Branched Endovascular Aortic Repair
Source: J Endovasc Ther. 2024 Nov 22;33(3):1354–62. doi: 10.1177/15266028241300005 (PMC13172123; doi:10.1177/15266028241300005)
Supplement: sj-docx-3-jet-10.1177_15266028241300005 – Supplemental material for Mid-Term Outcomes of the Viabahn Balloon-Expandable Endoprosthesis as Bridging Stent Graft for Fenestrated and Branched Endovascular Aortic Repair [file sj-docx-3-jet-10.1177_15266028241300005.docx]

**Supplementary table 3: Placement of VBX stent-graft in target vessels**

|  | | Number of VBX implanted | |  |
| --- | --- | --- | --- | --- |
|  |  | 1 | 2 | Total |
| Target vessel | CT | 41 | 3 | 44 |
|  | SMA | 70^a^ | 3 | 73 |
|  | LRA | 69^b^ | 2 | 71 |
|  | RRA | 69 | 2 | 71 |
| Total number of target vessels | | 249 | 10 | 259 |

**Supplementary table 3:** Number of VBX stent-graft implanted as a bridging stent-graft per target vessel. ^a^ Two SMAs had an additional Advanta V12 stent-graft implanted. ^b^ One LRA had an additional Advanta V12 stent-graft implanted. CT: celiac trunk, SMA: superior mesenteric artery, LRA: left renal artery, RRA: right renal artery.
