# Supplementary material for: Mid-Term Outcomes of the Viabahn Balloon-Expandable Endoprosthesis as Bridging Stent Graft for Fenestrated and Branched Endovascular Aortic Repair
Source: J Endovasc Ther. 2024 Nov 22;33(3):1354–62. doi: 10.1177/15266028241300005 (PMC13172123; doi:10.1177/15266028241300005)
Supplement: sj-docx-4-jet-10.1177_15266028241300005 – Supplemental material for Mid-Term Outcomes of the Viabahn Balloon-Expandable Endoprosthesis as Bridging Stent Graft for Fenestrated and Branched Endovascular Aortic Repair [file sj-docx-4-jet-10.1177_15266028241300005.docx]

**Supplementary table 4: Femoral and brachial access per target vessel design**

|  |  | Femoral | Brachial | Total |
| --- | --- | --- | --- | --- |
| Target vessel design | Outer-branch | 78 | 4 | 82 |
|  | Inner-branch | 25 | 45 | 70 |
|  | Fenestration | 104 | 3 | 107 |
| Total |  | 207 | 52 | 259 |

**Supplementary table 4:** Number of target vessels with femoral and brachial approach, stratified by target vessel design.
